# Kar9 symmetry breaking alone is insufficient to ensure spindle alignment

**DOI:** 10.1038/s41598-021-83136-w

**Published:** 2021-02-19

**Authors:** Miram Meziane, Rachel Genthial, Jackie Vogel

**Affiliations:** Department of Biology, McGill University, Montreal, QC H3G 0B1 Canada

**Keywords:** Biophysics, Cell biology

## Abstract

Spindle positioning must be tightly regulated to ensure asymmetric cell divisions are successful. In budding yeast, spindle positioning is mediated by the asymmetric localization of microtubule + end tracking protein Kar9. Kar9 asymmetry is believed to be essential for spindle alignment. However, the temporal correlation between symmetry breaking and spindle alignment has not been measured. Here, we establish a method of quantifying Kar9 symmetry breaking and find that Kar9 asymmetry is not well coupled with stable spindle alignment. We report the spindles are not aligned in the majority of asymmetric cells. Rather, stable alignment is correlated with Kar9 residence in the bud, regardless of symmetry state. Our findings suggest that Kar9 asymmetry alone is insufficient for stable alignment and reveal a possible role for Swe1 in regulating Kar9 residence in the bud.

## Introduction

Asymmetric cell divisions are common in nature, mediating essential processes like development and regeneration. Asymmetric cell divisions occur when cells resulting from the division differ in size and/or cell fate. Crucial to the success of an asymmetric cell division is the precise regulation of spindle positioning. In this context, the spindle can be positioned off center such that the cytokinesis event produces daughters of different sizes. Additionally, cytoplasmic factors may be differentially distributed such that daughters differ in cell fate. Cortical cues are translated into spindle motions to ensure that the daughters inherit the appropriate factors. Both cases are illustrated in the first cell division of the *C. elegans* embryo. The polarity of the cortex leads to the spindle being positioned off center toward the posterior, where P-granules are also clustered^1^. The division results in a large somatic cell, and a smaller cell that contains the P-granules, which will develop into the germline of the worm^2^.

The budding yeast *S. cerevisiae* also divides asymmetrically. Growth factors must be concentrated at a prespecified bud site, to ensure emergence and growth of the bud. This requires the reorganization of the actin cytoskeleton to allow polarized transport of cargoes^3^. Specifically, the long clusters of actin filaments known as actin cables originate from the bud tip and bud neck through the localization of formins Bni1 and Bnr1 respectively^4^. This ensures that cables emanate from the bud, allowing polarized transport of cargoes via the type V myosin Myo2^3^.

As the bud neck is the future plane of cytokinesis, the metaphase spindle must position itself relative to the bud neck to ensure proper segregation of chromosomes into the bud and mother compartments in anaphase. Moreover, the spindle itself has asymmetric properties. The spindle pole bodies, which are equivalent to centrosomes, undergo conservative replication, resulting in an old (pre-existing) and new (newly synthesized) spindle pole^5^. It is the old spindle pole that is preferentially oriented toward and inherited by the bud in 98% of cases^6^.

A unifying feature of spindle positioning is the coupling of cortical cues and spindle movements. This crucial element is mediated by spindle pole nucleated microtubules (MTs) that project into the cytoplasm known as astral MTs (aMTs). The aMTs exert forces on the spindle pole bodies to correctly position the spindle through their + end interactions with MT associated proteins (MAPs), MT + end tracking proteins (+ TIPs), cortical proteins and motor proteins. The proteins at the cortex allow the aMTs, and by extension the spindle pole bodies, to read polarity cues and translate them into spindle pole motions leading to the alignment of the spindle axis with the polarity axis of the cell^2^.

In budding yeast, the + TIP Kar9 is responsible for aligning the spindle and orienting the old spindle pole toward the bud. Kar9 interacts with the cargo-binding domain of the type V Myosin Myo2, an interaction that directs Kar9 to the bud tip^7^. Whether this interaction is subject to spatiotemporal regulation remains unclear. Evidence suggests that Kar9 may interact with Myo2 in both mother and bud compartments: aMT sweeping in the mother compartment has been used as evidence of Myo2-dependent transport^8^. Once bound, the Kar9-Myo2 interaction effectively translates the polarity of the cortex into spindle pole movements via the processive motion of the Kar9-Myo2 complex toward the bud tip^7^. Only once Kar9 enters the bud is the spindle expected to align along the polarity axis. This suggests a potential role for spatiotemporal regulation that could promote persistent Kar9-Myo2 interaction in the bud, thus ensuring effective spindle alignment.

**Figure 1:**
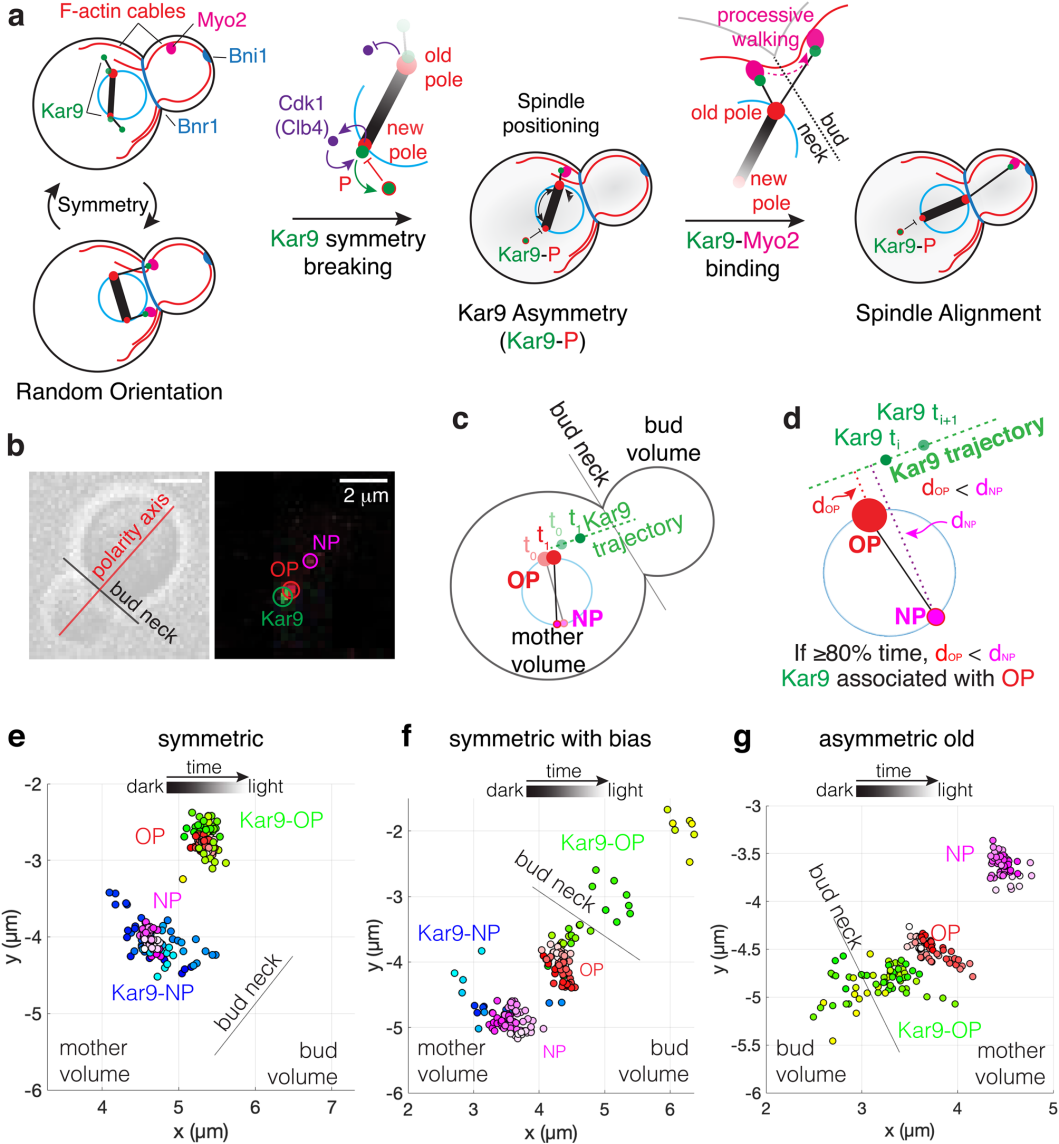
A trajectory-based method of Kar9-pole association. (**a**) After bipolar spindle formation, the spindle is randomly oriented. Both spindle poles and associated aMTs are decorated with Kar9. In this regime, Kar9 from both poles may interact with Myo2, thus causing both poles to be directed toward the bud, resulting in misalignment. Symmetry is broken by Cdk1/Clb4, where Kar9 at the new pole is targeted and phosphorylated by this complex. Once Kar9 asymmetry is achieved, the spindle is amenable to spindle positioning. This occurs through the processive walking of the Kar9-Myo2 complex toward the bud tip, resulting in spindle alignment. (**b**) Representative brightfield image used to determine bud neck and polarity axis and representative fluorescent image (max-projected). Old and new pole are differentiated based on fluorescence intensity. Scale bar 2 μm. (**c**,**d**) Graphical description of trajectory method. (**e**) An example of a symmetric state: Kar9 associates with both old and new pole over the entire acquisition. (**f**) An example of a symmetric state with bias: these cells fluctuate between symmetry and asymmetry. (**g**) An example of an asymmetric old cell: Kar9 associates exclusively with a single pole (old pole) over the entire acquisition.

Kar9 may interact with both the old and new spindle poles and their associated aMTs. If this is the case, both spindle poles may be directed toward the bud, resulting in spindle misalignment^8^. Therefore, it is believed that Kar9 symmetry breaking and accumulation to the old spindle pole is required to effectively align the spindle (Fig. 1a). Kar9 symmetry breaking is subject to regulation by Cdk1/Clb4. Kar9 is phosphorylated in vivo by the Cdk1/Clb4 complex at two sites (S197, S496). By blocking Cdk1 phosphorylation at these sites through S > A substitutions (S197A-S496A; *kar9-AA*), a significant increase in Kar9 symmetry was found in the cell population^8–10^. The phosphorylation is believed to decrease Kar9 binding affinity to Bim1 on the new spindle pole and as a consequence Kar9 binds to the old spindle pole associated aMTs^8^ (Fig. 1a).

The model shown in Fig. 1a is based on previous findings that demonstrated that the regulation of Kar9 asymmetry is essential for spindle alignment. While the temporal coupling between symmetry breaking and spindle alignment is expected to be variable, it is assumed that (1) once Kar9 is asymmetric the likelihood that Kar9 will enter the bud increases significantly and (2) entry of Kar9 into the bud is highly correlated with spindle alignment. However, the relationship between Kar9 symmetry breaking, Kar9 residence in the bud compartment, and spindle alignment have not been studied as a dynamic process, therefore the temporal correlation between Kar9 symmetry breaking and residence in the bud is not clear. In the present study, we explore precisely this question by taking fast acquisitions of pre-anaphase spindles, and correlating spindle alignment with Kar9 symmetry state and Kar9 residence in the bud compartment. We developed a method to reliably associate Kar9 foci with the spindle pole (old or new) of origin to determine symmetry states. This analysis revealed that there are two principle states; a symmetric state that is correlated with short metaphase spindles and early metaphase and an asymmetric state that is correlated with increasing length and time in metaphase. Moreover, in the symmetric state, we find that the fluctuation of Kar9 localization and abundance on the two spindle poles are not significant parameters for spindle alignment. We perturbed the symmetry breaking pathway using mutations and were able to explore the effects of Kar9 symmetry breaking on spindle alignment. Upon examination we found that the majority of asymmetric spindles did not reach steady state alignment. Further investigation showed that steady state alignment (perfect alignment) was better correlated with Kar9 residence time in the bud rather than symmetry state. Moreover, our findings suggest that spindle alignment requires regulation that retains Kar9 in the bud.

## Results

### A trajectory-based method of Kar9-pole association identifies symmetric and asymmetric states

Previous determination of Kar9 symmetry required aMT labelling to visually assign the symmetry state of each cell^8^. However, Kar9 symmetry state can be determined in the absence of aMT contouring using Kar9 trajectories by employing + TIP tracking to trace aMT dynamics at short timescales. Specifically, two consecutive Kar9 spots in a track can define a line in 3D tracing the motion of the dynamic aMT + end, eliminating the need for aMT labelling. This method relies on the fact that over the course of a short time step (5 s), the spindle poles move slowly in comparison to highly dynamic aMT + ends and assumes the majority of aMTs contours are linear in this regime. Therefore, two consecutive Kar9 spots should trace the position of the MT + end in relation to the spindle pole of origin that has remained nearly stationary in comparison. Since this method requires high temporal resolution, only a snapshot of the pre-anaphase spindle alignment process is captured, which normally takes 20–30 min to achieve. Instead of tracing symmetry breaking over time, symmetry states over the course of spindle alignment are captured and correlated with alignment of the spindle axis to the polarity axis as well as spindle length.

Analysis methods are detailed in the Methods section. In brief, yeast expressing Kar9-mNeonGreen and spindle pole body reporter Spc42-mKate2 were imaged every 5 s for 5 min. Brightfield images were used to establish the axes of the cell (Fig. 1b). Fluorescent images were used to track objects in three-dimensions (3D) with high precision by fitting signal intensity to a 3D Gaussian (implemented with TrackMate^11^) (Fig. 1b). Spindle pole tracks were used to determine length and orientation of the spindle. Kar9 tracks were used to determine the pole of origin. Consecutive coordinates in the track were used to define a line in 3D, and the pole closest to the line was recorded. The operation was repeated for every consecutive pair, and the pole closest to the track over 80% of the time was deemed the pole of origin (Fig. 1c,d). Symmetry was then determined based on Kar9-pole association. Spindles were classified as asymmetric when all Kar9 tracks pointed to a single pole (Fig. 1g). Conversely, spindles were classified as symmetric when a fraction of the tracks pointed to the old pole and others the new pole (Fig. 1e,f).

**Figure 2:**
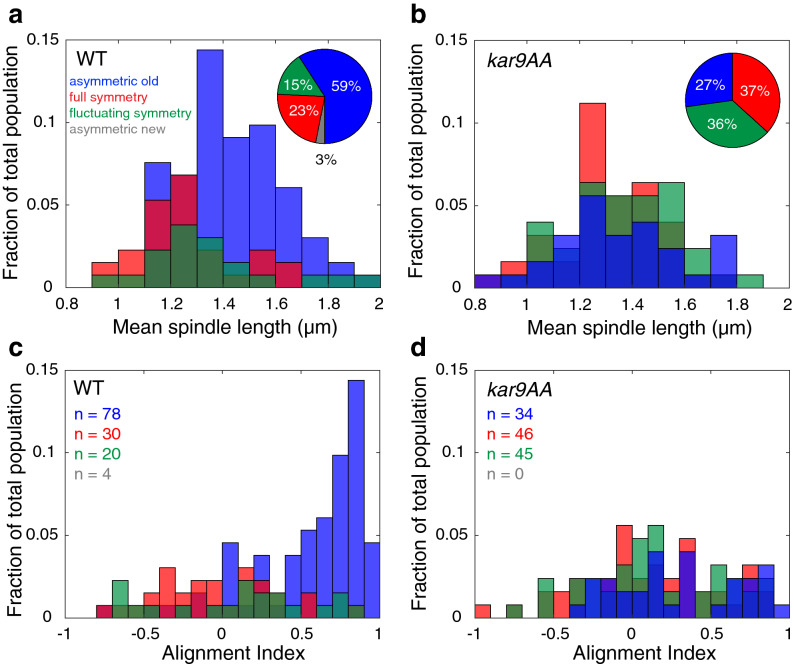
Full and fluctuating symmetric classes are similar states. (**a**,**b**, inset) Proportion of asymmetric old, full symmetry, fluctuating symmetry and asymmetric new spindles in WT (**a**), *kar9-AA* (**b**) populations. (**b**, inset) Blocking Cdk1 regulation of Kar9 effectively increases the proportion of symmetric (full and fluctuating) cells in the population. (**a**) Symmetry breaking is correlated with increasing spindle length, and approach to anaphase in WT cells. (**b**) Symmetry breaking occurs at random in the *kar9-AA* population. (**c**) Asymmetric WT cells are biased toward spindle alignment. (**d**) *kar9-AA* asymmetric spindles are defective in alignment. In both WT and *kar9-AA* populations, spindles with full or fluctuating symmetry are broadly and uniformly distributed across the range of AIs.

Upon examination of wild type (WT) cells, we found a large heterogeneity in symmetry states. Kar9 exhibited a variety of behaviours, such as exclusive association with the old or new pole (asymmetric old or asymmetric new respectively, Fig. 1g) or association with both poles over the entire acquisition (symmetric, Fig. 1e). Furthermore, a fraction (20 of 132) of spindles were observed to fluctuate between symmetry and asymmetry (Fig. 1f). To validate the method used in the present study, frequencies of Kar9 symmetry and asymmetry in WT and *kar9-AA* populations were examined and compared to previously published work. As *kar9-AA* cells have previously been characterized to be overwhelmingly symmetric^8^, classification of the population through our method was expected to yield a similar frequency of symmetry. Indeed, the *kar9-AA* population was primarily symmetric (73%, 91 of 125 cells). As with WT symmetric cells, both full symmetry and fluctuating symmetry was observed in the *kar9-AA* mutant, with 37% (46 of 125 cells) of the population displaying full symmetry and 36% fluctuating symmetry (45 of 125 cells, Fig. 2b, inset). In comparison to the *kar9-AA* mutant, the WT population was primarily asymmetric (59%, 78 of 132 cells, Fig. 2a, inset). As the symmetric population in both WT and *kar9-AA* cells exhibits both full and fluctuating symmetry, this suggests these behaviors are characteristic of Kar9 dynamics prior to symmetry breaking.

### Diverse Kar9 symmetry states are not functionally distinct

Next, we sought to determine the timing of symmetry breaking. Absolute time spent is difficult to measure, as each cell progresses through the cell cycle at different rates. However, the pre-anaphase bipolar spindle grows at a relatively constant rate of 0.15 nm/s in WT cells and mutants used in this study (Supplementary Fig. S3), therefore mean spindle length can be used as a proxy for time. Short spindles (≈ 1 μm) represent cells that have just recently formed a bipolar spindle and increasing spindle length is proportional to increasing time spent in metaphase^12^. By correlating spindle length with symmetry state, we report that the majority of symmetric (both full and fluctuating) WT spindles were short, with a sharp drop at about 1.3 μm in length. The majority of asymmetric WT spindles were found at relatively longer spindle lengths (Fig. 2a). Therefore, symmetry is associated with early bipolar spindle formation, and symmetry breaking is proportional to increasing time.

In the absence of Cdk1 regulation of Kar9 via the *kar9-AA* mutation, symmetry breaking is expected to be uncoupled from progression to the metaphase/anaphase transition, with symmetry breaking occurring at random. Indeed, our analysis revealed symmetry state to be uncoupled from spindle length. Instead, both fluctuating and fully symmetric populations, as well as asymmetric populations were similar: they were broadly distributed along the entire range of short spindle lengths, indicating a lack of regulation in the system (Fig. 2b).

To characterize the process of spindle alignment in the context of different symmetry states, we employed a previously developed method to describe 3D spindle alignment^13^. The 3D spindle coordinates are projected onto the polarity axis, resulting in a one-dimensional (1D) projected length. The 1D length is plotted versus the true 3D length to determine alignment. The spindle is aligned when 1D and 3D lengths are comparable. To simplify the analysis, we defined an alignment index (AI) as the mean ratio of 1D to 3D length for each spindle. A numerical value of AI ≈ 1 indicates proper alignment and orientation (old pole proximal to the bud). A numerical value of AI ≈ − 1 implies mis-oriented alignment (new pole proximal the bud). All other AI values ranging from − 1 to 1 designates various degrees of misalignment, where an AI ≈ 0 indicates the spindle is perpendicular to the polarity axis. Regardless of genotype, both full and fluctuating symmetry spindles were broadly distributed around an AI value of 0, indicating poor alignment (Fig. 2c,d). This is consistent with a regime in which either the new or old pole can be transiently directed towards the bud neck and is the expected outcome of the symmetric state (Fig. 1a). In contrast, the majority of WT asymmetric spindles were biased toward a high AI (Fig. 2c). The small fraction of asymmetric *kar9-AA* spindles showed variation in their alignment, with a broader distribution for AI relative to WT asymmetric spindles (Fig. 2d). All 3D spindle measures are consistent with 2D alignment measures (Supplementary Fig. S1).

**Figure 3:**
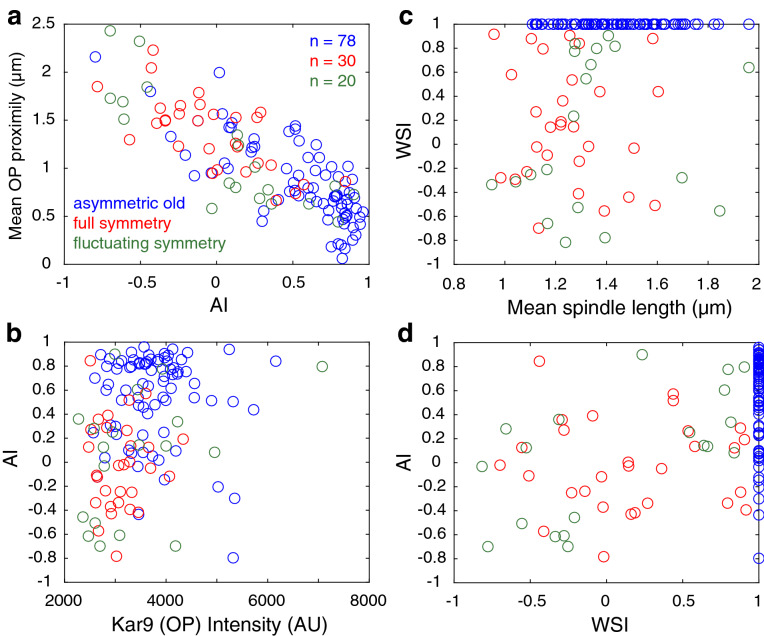
Kar9 bias to a given pole does not influence spindle alignment in wild-type cells. (**a**) Old pole (OP) proximity to the bud neck decreases as alignment index (AI) increases, irrespective of symmetry state. (**b**) Signal intensity of Kar9 associated with the old pole is distributed between 2000 and 4000 AU in most cells, and uncorrelated with symmetry state and alignment. (**c**) WSI, representative of Kar9 bias for a given pole, is insensitive to metaphase progression. (**d**) Spindle alignment is largely unaffected by Kar9 bias in symmetric cells.

In order to examine the functional relevance of the observed symmetry state diversity, we explored the relationship between various traditional parameters of interest with AI in WT cells. As expected, AI and proximity of the old pole to the bud neck were inversely proportional; as alignment is improved and AI approaches 1, the old pole approaches the bud neck, decreasing the distance between them. This trend was observed irrespective of symmetry state; however the majority of asymmetric old cells were more proximal to the bud neck than symmetric cells (Fig. 3a).

Kar9 intensity at the old pole is often explored in relation to spindle alignment and symmetry breaking. We examined whether Kar9 signal intensity could accurately predict spindle alignment. Regardless of symmetry state, Kar9 signal intensity remained within a 2000–4000 AU range for nearly all cells, suggesting that Kar9 intensity at the old pole has a negligible effect on spindle alignment (Fig. 3b).

Next, we questioned whether Kar9 preference to one pole over the other influenced spindle alignment. To do so, we defined the weighted symmetry index (WSI), a parameter which describes integrated intensity of Kar9 signal and the time that signal occupies each pole (detailed in Methods section). Given this definition, the WSI of asymmetric old spindles is + 1, and that of asymmetric new spindles is − 1. The WSI then reflects the bias of Kar9 toward a given pole in symmetric cells. For example, a fully symmetric cell with more Kar9 accumulated on the old pole will have a positive, non-zero WSI, while cells with more Kar9 on the new pole will have a negative, non-zero WSI. Cells with fluctuating symmetry in which an occupancy bias is already present, will deviate toward + 1 or − 1 depending on the intensity of the Kar9 signal at each pole.

Using this measure, we asked whether WSI was sensitive to metaphase progression. No clear relationship emerged between spindle length and WSI as short symmetric spindles displayed a wide range of WSI values. This suggests that there is no transition from one state to another as metaphase progresses (Fig. 3c).

Finally, we asked whether Kar9 bias as represented by the WSI measure was correlated with 3D spindle alignment. This analysis revealed little to no correlation between the two; cells at a given WSI range still sampled the majority of the allowable AI space (Fig. 3d).

Given the results shown in Figs. 2 and 3, we conclude that the relative intensity of Kar9 on the old pole has a negligible effect on the spindle alignment process. Several parameters show that full and fluctuating symmetric spindles are similar to each other, and distinct from asymmetry. Our results show that even a small amount of Kar9 at one pole may be sufficient for Myo2 directed transport of the aMT + end to the bud, consistent with previous reports^14^. Full and fluctuating symmetric spindles are nearly identical in spindle length, WSI and alignment distributions. Therefore, for the remainder of this study we chose to classify all cells displaying Kar9 association with both poles, irrespective of symmetry index and fluorescence distribution as symmetric, where symmetry breaking had not yet occurred.

### Swe1 is required to temporally couple Kar9 asymmetry to perfect alignment

**Figure 4:**
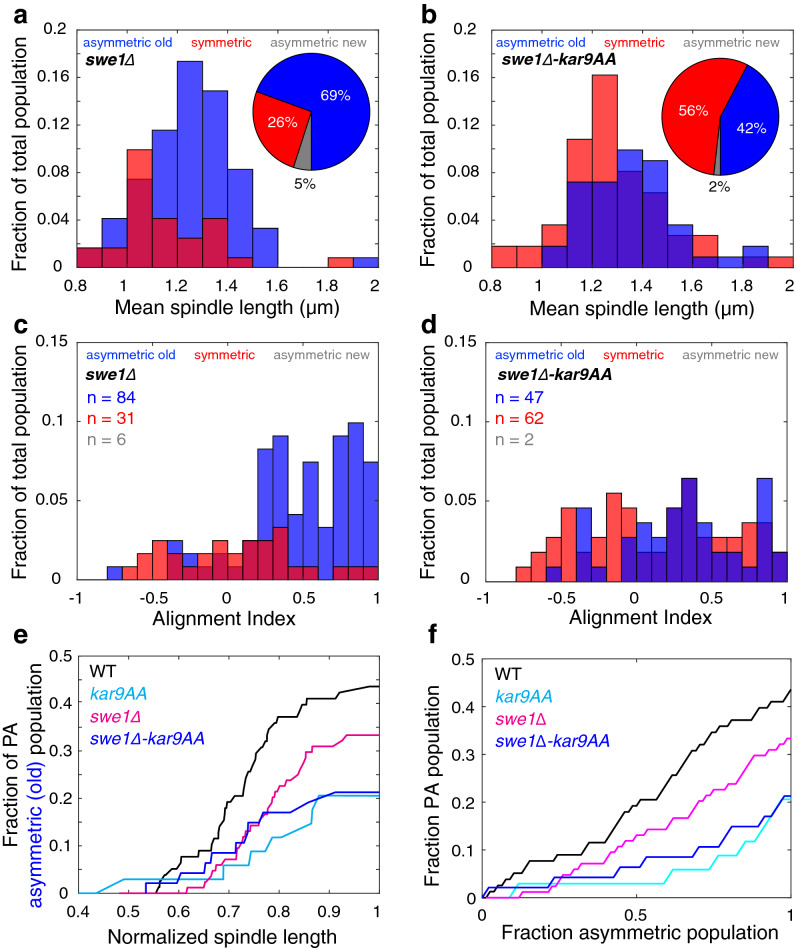
Swe1 acts to regulate symmetry breaking independently of the Cdk1-Kar9 pathway. (**a**, inset) *swe1∆* cells display increased asymmetry in the population relative to WT. (**a**) Deletion of Swe1 results in early symmetry breaking. (**b**, inset). Deletion of Swe1 partially rescues the symmetry breaking defect in the *kar9-AA* population. (**b**) Symmetry breaking occurs at random in the double mutant. (**c**) Asymmetric *swe1∆* cells are biased toward alignment but are less efficient that WT asymmetric cells. (**d**) Asymmetric cells in the double mutant population are broadly distributed along a large range of AI values indicating a poor ability to align themselves. (**e**) Cumulative percentage of PA spindles in asymmetric populations as a function of increasing spindle length, which is a proxy for time spent in metaphase. Over time, WT asymmetric spindles achieve perfect alignment more efficiently than the *swe1∆* mutant and *swe1∆; kar9-AA* double mutant. A small population of PA asymmetric *kar9-AA* cells emerges later in metaphase. (**f**) The correlation between perfect alignment and asymmetry is reduced in the *swe1∆* mutant relative to WT. The *swe1∆; kar9-AA* double mutant behaves similarly to *kar9-AA*.

We next decoupled Cdk1 inhibition of Kar9 from cell cycle progression by deleting the Wee1 ortholog Swe1^15^. Swe1 inhibits both early (Clb3,4) and late (Clb1,2) M-phase forms of Cdk1 in budding yeast^15,16^. Therefore, M-phase Cdk1 activity is expected to be unrestrained in the *swe1∆* mutant with symmetry breaking occurring earlier than in WT cells. *swe1∆* cells spanned a smaller range of pre-anaphase spindle lengths, consistent with the role of Swe1 in mediating the metaphase-to-anaphase transition^12,17^. The difference in spindle length may also be attributed to the smaller size of the cells. As expected, we observed an increase in the number of asymmetric cells in the population (69%, 84 of 121 cells, Fig. 4a, inset). Importantly, we observed a subset of Kar9 asymmetric cells in the population at short spindle lengths that was not observed in WT asymmetric cells. This suggests that symmetry breaking occurs earlier in the absence of Swe1 (Fig. 4a).

To determine if the phenotype observed was indeed due to unrestrained Cdk1 activity and not from other Swe1 targets^18^, we generated a *swe1∆; kar9-AA* double mutant. Surprisingly, deleting Swe1 in the *kar9-AA* background influenced symmetry breaking, as we observed an increase in the number of asymmetric cells in this population (42%, 47 of 111 cells, Fig. 4b, inset). Despite an increase in the frequency of asymmetry in the population, the timing of symmetry breaking was similar to that of the *kar9-AA* mutant, as both asymmetric and symmetric populations broadly spanned the entire range of spindle lengths (Fig. 4b).

By employing the *swe1∆* mutant, we were able to examine if early symmetry breaking translates into early spindle alignment. Remarkably, while *swe1∆* cells broke symmetry earlier, the asymmetric spindles were less efficient in alignment relative to their WT counterparts (mean AI = 0.49, relative to 0.55 for asymmetric WT cells, Fig. 4c). Asymmetric *swe1∆; kar9-AA* spindles are defective in alignment similar to the *kar9-AA* population (Fig. 4d). Symmetric spindles in both regimes failed to align themselves (Fig. 4c,d), consistent with their WT and *kar9-AA* counterparts.

In order to better characterize spindle alignment in these populations, we classified spindles as perfectly aligned (PA) or not aligned (NA) based on alignment index. If the absolute value of the AI is greater than 0.75, the spindles are perfectly aligned (AI ≥ 0.75, PA toward the old pole; AI ≤ − 0.75, PA toward the new pole). All other spindles are classified as NA. This threshold represents the quality of the spindle’s alignment. If the spindle is oriented such that one pole is able to enter the bud if the cell were to undergo anaphase at that point, the spindle is PA. However, if the orientation of the spindle does not allow for spindle pole body entry upon anaphase, the spindle is classified as NA.

Using these categorizations, we unexpectedly found that the majority of asymmetric spindles were not perfectly aligned (44%, 34 of 78 cells, Fig. 4e), suggesting that perfect alignment is poorly correlated with asymmetry. Furthermore, perturbations in the symmetry breaking pathway decreased the frequency of PA spindles. This is especially notable in the *swe1∆* asymmetric population, where only 33% of spindles were PA, despite an overall increase in the frequency of asymmetric spindles (28 of 84 cells, Fig. 4e). Similarly, double mutant spindles displayed similar PA frequencies to *kar9-AA*, despite having a larger population of asymmetric spindles (21%, 10 of 47 cells, Fig. 4e). Moreover, there were a subset of symmetric spindles that were perfectly aligned, supporting the observed poor correlation between symmetry breaking and spindle alignment (Supplementary Fig. S2). Finally, we directly related the relationship between spindle alignment and asymmetry and confirmed that the fraction of cells with perfectly aligned spindles is reduced in the *swe1∆* and *swe1∆; kar9-AA* asymmetric population relative to WT (Fig. 4f). Thus, in the absence of Swe1 spindle alignment is defective independent of Kar9 asymmetry.

The 5-min acquisition length does not allow for the observation of the entire spindle alignment process. However, we can estimate when perfect alignment is achieved in the population by using spindle length as a proxy for time. Since *swe1∆* spindles undergo the metaphase-to-anaphase transition at shorter spindle lengths^12^, spindle length is normalized to the mean spindle length prior to the onset of anaphase. To ensure spindle growth was comparable between strains, we measured the mean spindle growth over the 5-min acquisition for each population. Indeed, all three strains demonstrated similar growth distributions (with a mean of 0.15 ± 0.68, 0.26 ± 0.77, 0.09 ± 0.59 and 0.36 ± 0.70 nm/s for WT, *kar9-AA*, *swe1∆* and *swe1∆; kar9-AA* respectively). Furthermore, the computed spindle growth was independent of initial spindle length (Supplementary Fig. S3).

WT asymmetric spindles steadily became more perfectly aligned as spindle length increased, while WT symmetric ones did not (Fig. 4e). While both *kar9-AA* and *swe1∆; kar9-AA* populations gradually became more aligned over time, fewer asymmetric cells in these backgrounds achieved perfect alignment at a given spindle length relative to WT asymmetric cells (Fig. 4e). Asymmetric spindles in the *swe1∆* population achieved perfect alignment as metaphase progressed more efficiently than *kar9-AA* cells but less efficiently than the WT (Fig. 4e). Altogether, our data suggests a disparity between symmetry breaking and alignment: while cells are asymmetric, the majority of spindles are not aligned. Furthermore, increasing and decreasing rates of symmetry breaking in the population via the *swe1∆* and *kar9-AA* mutants does not proportionately affect alignment, rather they are both detrimental to reaching perfect alignment.

### Kar9 residence time in the bud predicts alignment efficiency

**Figure 5:**
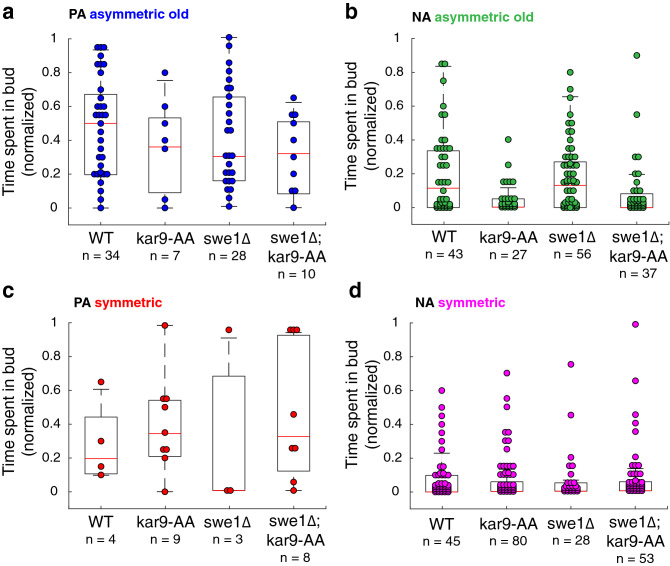
Spindle alignment is correlated with Kar9 residence time in the bud. Number of time points spent in bud normalized to length of the acquisition for (**a**) PA-asymmetric spindles, (**b**) NA-asymmetric spindles, (**c**) PA-symmetric spindles and (**d**) NA-symmetric spindle. In PA-asymmetric spindles, Kar9 spends more time in the bud than NA-asymmetric spindles or PA and NA symmetric spindles. For both PA and NA-asymmetric spindles, the *kar9-AA* and *swe1∆* mutations reduce Kar9 residence time in the bud.

Evidence clearly suggests that perfect alignment and asymmetry are not well correlated, prompting us to investigate what else was needed to achieve perfect alignment. Therefore, we examined Kar9 localization to the mother and bud compartments (Fig. 5). We defined Kar9 residence time as the fraction of time points of the total acquisition Kar9 localized to the bud. As the residence times for each condition are not normally distributed, we focused on the median for each and computed the absolute median distribution (Table 1). Residence times of WT PA asymmetric spindles (0.5000 ± 0.2439, n = 34) are increased relative to *swe1∆* PA asymmetric spindles (0.2951 ± 0.2523, n = 28) and all NA asymmetric spindles. This was also true in strains with *kar9-AA* mutations (Fig. 5a,b; Table 1), suggesting Kar9 residence in the bud to be an important aspect of spindle alignment independent of symmetry breaking. Furthermore, the extent of Kar9 residence time in the bud was consistent with alignment trends of WT, *kar9-AA*, *swe1∆* and *swe1∆; kar9-AA* populations (Fig. 5a,b; Table 1). While perfect alignment is correlated with time spent in the bud, Kar9 of mutant populations spent less time in the bud relative to their WT counterparts, which may explain why these populations are less effective at spindle alignment. Curiously, symmetric PA spindles in *kar9-AA* and *swe1∆; kar9-AA* populations span a broader range of residence times relative to the WT (Fig. 5c). This may be due to compensation by dynein-mediated spindle positioning in the *kar9-AA* mutant, with Kar9 dwell time increasing due to aMTs engaged with dynein at the bud cortex. As expected, Kar9 residence time was lowest in NA-symmetric spindles irrespective of strain background (Fig. 5d; Table 1).

**Table 1:** Median Kar9 residence time in bud (normalized).

Strain	Asymmetric old		Symmetric	
	PA	NA	PA	NA
WT (126 cells)	0.5000 ± 0.2439 n = 34	0.1148 ± 0.1932 n = 43	0.1967 ± 0.1680 n = 4	0 ± 0.1036 n = 45
*kar9AA* (123 cells)	0.3607 ± 0.2074 n = 7	0 ± 0.0596 n = 27	0.3443 ± 0.2170 n = 9	0 ± 0.0788 n = 80
*swe1∆* (115 cells)	0.2951 ± 0.2523 n = 28	0.1311 ± 0.1528 n = 56	0 ± 0.4007 n = 3	0 ± 0.0930 n = 28
*swe1∆; kar9AA* (108 cells)	0.3197 ± 0.1902 n = 10	0 ± 0.1050 n = 37	0.3197 ± 0.3514 n = 8	0 ± 0.1036 n = 53

Median ± absolute median distribution.

**Figure 6:**
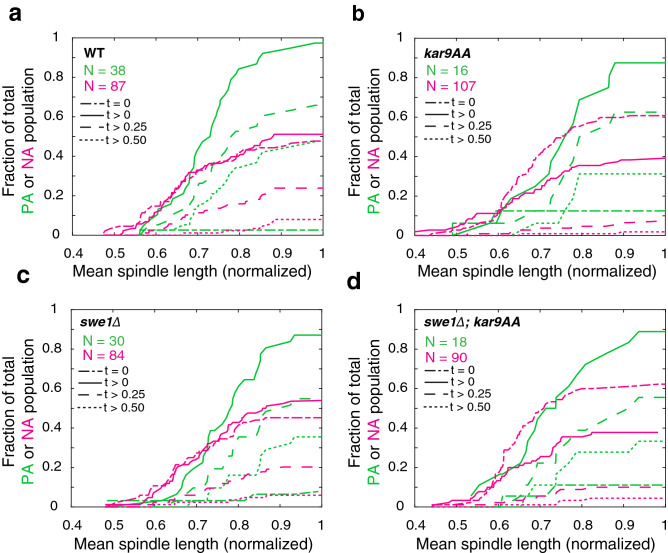
Deletion of Swe1 delays spindle alignment by reducing Kar9 residence time in the bud (**a**) The number of WT spindles displaying non-zero residence times increases with spindle length. (**b**) Similarly, residence time in the bud is correlated with metaphase progression in *kar9-AA* cells. (**c**) *swe1∆* consistently display delays in the accumulation of non-zero residence times, irrespective of the state of alignment. (**d**) *swe1∆; kar9-AA* behave like the *kar9-AA* population.

Next, we asked whether Kar9 bud entry was correlated with metaphase progression, irrespective of symmetry state. To do so, we pooled cells into PA and NA classes, and examined the fraction of cells with Kar9 residence times equal to or above a given residence time threshold. At this stage it is difficult to determine a biological residence time threshold, therefore we explored trends for a variety of thresholds (t = 0, t > 0, 0.25, 0.50, Fig. 6a–d). Spindle length was normalized to the mean spindle length prior to anaphase onset^12^ to facilitate comparison between strains.

At a normalized length of ≈ 0.55 (1.1 μm), WT PA spindles with non-zero residence times began to accumulate. The large majority of PA spindles with non-zero residence times spent over half of the time in the bud (Fig. 6a). Importantly, only one PA spindle displayed a residence time of zero, illustrating the importance of Kar9 dwelling in the bud. Conversely, WT NA spindles of zero and non-zero residence times accumulated similarly as metaphase progressed. Less than half resided in the bud for a quarter of the acquisition, and only began to accumulate later in metaphase (normalized length of ≈ 0.55, 1.1 μm, similar to PA spindles). Unlike PA spindles, few NA spindles displayed residence times exceeding half the acquisition. These events largely occurred late in metaphase (normalized length ≈ 0.85, 1.7 μm) (Fig. 6a). Notably, at a normalized length of ≈ 0.7 (1.4 μm), the number of PA spindles with non-zero residence times began to accumulate more rapidly. Meanwhile, NA spindles with non-zero residence times became more infrequent at the normalized length of ≈ 0.75 (1.5 μm) (Fig. 6a). This suggests a potential transition from random Kar9 bud entry to Kar9 retention within the bud, leading to perfect alignment.

Similarly, *kar9-AA* PA spindles with non-zero residence times began to accumulate at a normalized length of ≈ 0.5 (1 μm). In contrast with the WT, only one third of *kar9-AA* PA spindles displayed residence times exceeding half of the acquisition (5 of 16). These were also delayed with respect to the WT, beginning their accumulation at a normalized length of ≈ 0.7 (1.4 μm, relative to 0.55 in WT PA cells) (Fig. 6b). *kar9-AA* NA spindles exhibited similar patterns to that of WT NA spindles; shifted similarly in time and accumulating earlier than *kar9-AA* PA spindles (Fig. 6b).

As *swe1∆* cells were less efficient in alignment, we asked whether the mutant influenced alignment efficiency by altering Kar9 retention within the bud. *swe1∆* PA spindles with non-zero residence times began accumulating at a normalized length of ≈ 0.65 (1.11 μm), delayed relative to the WT which began its accumulation at a normalized length of 0.55. The separation between residence time thresholds in PA spindles was accentuated in *swe1∆* cells relative to the WT (Table 1). Furthermore, zero and non-zero residence times in NA spindles of WT and *kar9-AA* accumulated concurrently, however *swe1∆* NA spindles were delayed in achieving non-zero residence times (Fig. 6c). The consistent shifts toward longer normalized lengths observed in *swe1∆* cells suggests that Swe1 may promote the retention of Kar9 in the bud, thus facilitating spindle alignment.

Finally, we explored Kar9 retention in the bud in the *swe1∆; kar9-AA* double mutant. Like WT and *kar9-AA* strains, PA spindles with non-zero dwell times started to accumulate at a normalized length of 0.5 (1.0 μm). Moreover, the proportion of cells and timing of residence times exceeding 25% and 50% of the acquisition length most closely resembled the trends observed in the *kar9-AA* population. This suggests that delayed dwelling in *swe1∆* is restored by the *kar9-AA* mutation. The majority of NA spindles showed dwell times of zero, the rest had short dwell times, very few exceeding 25% of the acquisition, consistent with NA behavior in the WT (Fig. 6d).

## Discussion

In this study, we investigated the extent to which Kar9 symmetry breaking and stable spindle alignment (perfect alignment) are temporally correlated. By developing a method to dynamically assay Kar9 symmetry breaking, we were able to scrutinize its relation to spindle alignment as a function of spindle length. The spindle elongates at a relatively constant rate of 0.15 nm/s during metaphase (Supplementary Fig. S3) therefore spindle length serves as a proxy for time spent in metaphase. We were surprised to find a poor correlation between symmetry breaking and perfect alignment. The low number of WT asymmetric cells displaying perfect alignment suggests that symmetry breaking does not rapidly align the spindle (Fig. 4). To further examine the coupling between Kar9 symmetry breaking and spindle alignment, we manipulated the timing of Kar9 symmetry breaking using genetic perturbations known to inhibit Kar9 asymmetry (*kar9-AA*) or increase Cdk1 activity in metaphase (*swe1∆*). While frequencies of symmetry/asymmetry in the mutant populations were altered by the imposed genetic perturbations, frequencies of perfect alignment were not proportionally affected. Notably, *swe1∆* cells broke symmetry earlier yet produced fewer perfectly aligned spindles; such cells were delayed in achieving perfect alignment. Furthermore, Kar9 localization and persistence in the bud compartment was correlated with stable spindle alignment. This strongly suggests that Kar9 retention within the bud is far more important in achieving perfect spindle alignment than symmetry breaking itself. By examining the effects of *kar9-AA* and *swe1∆* on Kar9-bud localization, we identified delayed retention of Kar9 in *swe1∆* cells, thus highlighting Swe1 as a potential mediator of Kar9-bud localization and spindle alignment. Finally, partial rescue of Kar9 asymmetry in the *swe1∆; kar9-AA* suggests that Swe1 may mediate Kar9 symmetry breaking independently of the Cdk1-Kar9 pathway.

Kar9 displays a variety of behaviors from persistent association to one or both poles (asymmetry or full symmetry respectively) to varying degrees of fluctuating symmetry. Moreover, Kar9 can be differentially distributed on both poles. It is currently thought that these behaviors each affect the spindle alignment process. We investigated the trends in alignment of full and fluctuating symmetric spindles and report no significant difference between the two (Fig. 2). We further explored this by defining a weighted symmetry index that quantitatively described the fluorescence intensity and symmetric distribution of Kar9 over the course of the acquisition. Consistently, spindle alignment was largely insensitive to WSI (Fig. 3). Together, this suggests that a simple two state description of symmetry and asymmetry is sufficient to describe spindle alignment with respect to symmetry breaking.

The consequences of the imposed perturbations on the symmetry breaking pathway suggest that Kar9 asymmetry and spindle alignment are partially independently regulated. The number of perfectly aligned spindles in the WT asymmetric class may simply be an indication of the inherent efficiency of the system: reaching alignment takes time, therefore only a subset of cells in the unsynchronized population achieved it. If stable alignment is a consequence of symmetry breaking, and not an independent process, frequencies of perfect alignment in asymmetric populations should be comparable, regardless of genotype. The *kar9-AA* and *swe1∆* mutants altered symmetry distributions, making a population more or less symmetric respectively. However, asymmetric *kar9-AA* and *swe1∆* cells both displayed decreased alignment efficiency with respect to the WT. This suggests that these mutations influence alignment in addition to and independently of symmetry breaking, and the poor correlation between symmetry breaking and alignment is due to differential regulation of the two processes.

Furthermore, spindle alignment may be subject to spatiotemporal regulation. Kar9 residence time in the bud was correlated with perfect alignment, regardless of symmetry state (Fig. 5; Table 1). This highlights bud entry as an important parameter in spindle alignment. The ability to retain Kar9 in the bud compartment was correlated with metaphase progression, occurring more efficiently after a normalized length of ≈ 0.75 (1.5 μm) in WT cells (Fig. 6). While our data implies spatiotemporal regulation of spindle alignment, further quantification of Kar9-Myo2 interactions in space and time is required. Moreover, our data suggests that Swe1, either directly or indirectly, promotes Kar9 retention in the bud. We observed a decrease in the number of PA spindles displaying long (t > 0.5) residence times in the *swe1∆* population. Cells with long residence times were observed at longer spindle lengths and thus later in metaphase relative to the WT population, suggesting delayed retention in the bud. We speculate that Swe1 may be able to influence spindle alignment by phosphorylating Kar9 and/or Myo2, increasing their affinity to one another and thus retaining Kar9 in the bud.

By employing spatiotemporal regulation of alignment, the cell is able to reduce instances of spindle misalignment regardless of the Kar9 symmetry state. Assuming persistent Kar9-Myo2 attachments are not restricted in space or time, it is expected that symmetric spindles should display misalignment (AI ≈ 0). However, WT symmetric spindles are uniformly distributed in orientation indicating transient misalignment. Only when Kar9 symmetry is enforced in the *kar9-AA* mutant is the accumulation of static misaligned symmetric spindles observed in the population (Fig. 4b). In the WT population of cells with symmetric spindles, the likelihood that aMTs from both spindle poles enter the bud is low and thus imposing this spatial requirement on alignment the cell further ensures that only one spindle pole is directed toward the bud.

Altogether, we propose an alternate model to the pre-anaphase spindle alignment process, in which spatiotemporal regulation of symmetry breaking and spindle alignment is incorporated. Kar9 symmetry breaking and spindle alignment are dependent on activities occurring in distinct compartments: symmetry breaking in the mother compartment, and alignment in the bud compartment. Symmetry breaking, facilitated by Cdk1/Clb4 early in metaphase (≈ 1.3 μm), results in motions that favour the entry of Kar9 on aMTs to enter the bud. However, stable alignment requires persistent Kar9 localization in the bud, where Kar9-Myo2 interactions are potentially strengthened by Swe1 later in metaphase (≈ 1.5 μm). Due to the compartmentalization of these two processes, they likely occur partially independently: achieving asymmetry does not ensure spindle alignment, and perfect alignment can be observed in cells with Kar9 symmetry.

An unexpected outcome of this study was the partial rescue of the symmetry breaking defect in *kar9-AA* cells by *swe1∆*. Further study must be done to dissect the exact mechanism by which Swe1 may regulate symmetry breaking. One potential mechanism may be through its regulation of the SPB component Nud1. Swe1 has been shown to phosphorylate Nud1 in vivo, which is implicated in regulating the SPB inheritance^19^. Importantly, symmetry breaking is disrupted in mutant *nud1-44*^10,18^. Therefore, Swe1 may act through Nud1 to mediate the timing of Kar9 symmetry breaking.

It is interesting to contemplate the potential role of mitotic exit network (MEN) kinases in the regulation of stable spindle alignment. Asymmetric *kar9-AA* cells were the most deficient in perfect alignment (Fig. 4), consistent with the phospho-inhibiting alanine substitutions altering Kar9’s functionality independent of symmetry breaking. Notably, MEN kinases Dbf2/20 were shown to target Kar9, including S197, which is blocked by the *kar9-AA* mutation^10^. Therefore, MEN signaling may be involved in Kar9-mediated spindle alignment. Spindle positioning and the MEN are coupled by the localization of Tem1 activators and inhibitors in the bud and mother compartment respectively. This differential localization of activators and inhibitors creates activation zone in the bud and inhibitory zone in the mother^20^. Thus, the position of the spindle poles act as a sensor, as Tem1 remains inactive in the mother and only becomes active upon bud entry^20^. Perfect alignment, which is the stable positioning of the old spindle pole at the bud neck, is important for this sensor to work properly. Stable Kar9 asymmetry and perfect alignment is thus likely to be a result of signaling contributions by Cdk1, Swe1 and the MEN acting at the spindle poles and in the bud.

## Methods

### Strain construction

All strains used are derived from BY4741 and are listed in Supplementary Table S1. All fluorescently tagged proteins are expressed under their endogenous promoter.

### Growth conditions and live cell imaging

Yeast was grown overnight at 25 °C in 5 mL synthetic complete (SC). Liquid cultures were diluted to 0.2 OD and incubated 1.5–2 h at 25 °C to reach log phase before imaging. Imaging was performed at 25 °C on a custom built dual-camera spinning disk confocal microscope with the following components: a Leica DMi8 inverted microscope with Quorum Diskovery platform installed, 63×/1.47 numerical aperture objective, 50 μm pinhole spinning disk, two iXon Ultra 512 × 512 EMCCD cameras for synchronous acquisitions, 488 nm and 561 nm solid state lasers, and an MCL nano-view piezo stage with ASI three axis motorized stage controller. Imaging was mediated through MetaMorph acquisition software. 488 nm and 561 nm lasers at 25% and 35% power respectively were used to excite fluorophores simultaneously. 14 z-stacks of 300 nm step size and 55 ms exposure were taken every 5 s for a total of 5 min. Data is pooled from 3 to 4 independent experiments for each strain.

### Segmentation and tracking

Individual cells were manually segmented using BF images. The bud neck coordinates were determined by recording the coordinates of the inflection points of the bud neck. The polarity axis was determined as the axis perpendicular to the bud neck. The angle between the horizontal and the polarity axis was measured (0° to 360°): angle ranged from 0° to 180° if the bud faced upward and 180° to 360° if the bud faced downward. Bud size was measured as the distance between the bud tip and the midpoint of the bud neck. All measurements were taken using the basic Fiji toolbox.

Fluorescent images were tracked with high precision in 3D using Fiji plugin TrackMate^11^. A difference of gaussian filter (DoG) was used to detect fluorescent spots. A threshold intensity of 200 and 270 was used to detect mKate2 and mNeonGreen objects respectively. The simple Linear Assignment Problem (LAP) algorithm was used to link spots into tracks^21^. A distance threshold of 1 μm and 1.5 μm was input for Spc42 and Kar9 respectively. Gap-closing was allowed for Spc42 (max distance 1 μm, max length 2 frames), but not for Kar9. Track coordinates were exported for analysis in MATLAB.

### Kar9-pole association algorithm

A Kar9 trajectory vector was defined from the position of two consecutive Kar9 objects:

$$v=\left(\begin{array}{c}{v}_{1}\\ {v}_{2}\\ {v}_{3}\end{array}\right)=\left(\begin{array}{c}{x}_{t+1}-{x}_{t}\\ {y}_{t+1}-{y}_{t}\\ {z}_{t+1}-{z}_{t}\end{array}\right)$$

The vector was used to define a line tracing the trajectory of the motion:

$$\left(\begin{array}{c}x\\ y\\ z\end{array}\right)=\left(\begin{array}{c}{x}_{t}\\ {y}_{t}\\ {z}_{t}\end{array}\right)+\alpha \left(\begin{array}{c}{v}_{1}\\ {v}_{2}\\ {v}_{3}\end{array}\right)$$

The shortest distance between the line and each pole (*x*_*p*_*,y*_*p*_*,z*_*p*_) is perpendicular to the trajectory line. Therefore, the dot product of the two vectors must be zero:

$$\left(\begin{array}{c}{x}_{p}-x\\ {y}_{p}-y\\ {z}_{p}-z\end{array}\right)\cdot \left(\begin{array}{c}{v}_{1}\\ {v}_{2}\\ {v}_{3}\end{array}\right)=0$$

Expanding and substituting x, y and z for their respective values:

$$\left({x}_{p}-x\right){v}_{1}+\left({y}_{p}-y\right){v}_{2}+\left({z}_{p}-z\right){v}_{3}=0$$

$$\left({x}_{p}-{x}_{t}-\alpha {v}_{1}\right){v}_{1}+\left({y}_{p}-{y}_{t}-\alpha {v}_{2}\right){v}_{2}+\left({z}_{p}-{z}_{t}-\alpha {v}_{3}\right){v}_{3}=0$$

Solving for the unknown variable α:

$$\alpha =\frac{\left({x}_{p}-{x}_{t}\right){v}_{1}+\left({y}_{p}-{y}_{t}\right){v}_{2}+({z}_{p}-{z}_{t}){v}_{3}}{{{v}_{1}}^{2}+{{v}_{2}}^{2}+{{v}_{3}}^{2}}$$

The obtained value of α is used to determine the distance between each pole and the trajectory line:

$$d=\sqrt{{\left({x}_{p}-{x}_{t}-\alpha {v}_{1}\right)}^{2}+{\left({y}_{p}-{y}_{t}-\alpha {v}_{2}\right)}^{2}+{({z}_{p}-{z}_{t}-\alpha {v}_{3})}^{2}}$$

The closest pole is recorded. The measurement is repeated for each consecutive pair of Kar9 tracks. The pole most often proximal to the respective trajectory (80% of the time or more) is deemed the pole of origin. Asymmetric old (new) cells have all tracks associated with the old (new) pole. Symmetric cells have at least one track associated with the old pole and at least one track associated with the new pole. Tracks below the 80% threshold are discarded. Tracks of an entire cell are discarded if a track with less than 80% linkage interferes with ability to determine the symmetry state of the cell.

### Spindle growth measurements

Mean spindle growth over the entire acquisitions was computed as previously described^22^. In brief, a 3-point sliding average was computed, after which the data was fit to a line. The slope of the fitted line was used as the mean spindle growth.

### 2D alignment measures

The coordinates of the spindle poles is used to define a vector:

$${s}_{t}={\left(\begin{array}{c}{x}_{O}-{x}_{N}\\ {y}_{O}-{y}_{N}\end{array}\right)}_{t}={\left(\begin{array}{c}\Delta x\\\Delta y\end{array}\right)}_{t}$$

The angle (θ_t_) between the horizontal and the vector *s*_*t*_ is determined:

$${\theta }_{t}={\mathrm{tan}}^{-1}\left({\Delta y}/{\Delta x}\right)$$

Finally, the absolute difference between θ_t_ and the angle defined by the polarity axis (φ) is calculated:

$${\Phi }_{t}=|{\theta }_{t}-\phi |$$

This calculation is performed and recorded for every time point in the acquisition.

### 3D alignment measures

This method is adapted from Shulist et al., 2017^13^. A vector defined by the polarity axis is determined using the measured polarity axis angle (φ):

$$p= \left(\begin{array}{c}\mathrm{cos}\, \phi \\ \mathrm{sin}\, \phi \end{array}\right)$$

The 3D spindle pole body coordinates are first projected onto the XY plane simply by retaining only X and Y coordinates. They are then projected onto the polarity axis vector p. The magnitude of the projected, 1D vector is determined:

$${l}_{1D,t}={\left({x}_{O}-{x}_{N}\right)}_{t}\mathrm{cos}\,\phi +{({y}_{O}-{y}_{N})}_{t}\mathrm{sin}\,\phi $$

The calculation is repeated for each time point t in the acquisition. The alignment index is defined as the mean ratio of 1D to 3D lengths for the entire acquisition:

$$AI=mean\left(\frac{{l}_{1D}}{{l}_{3D}}\right)$$

### Weighted symmetry index

The weighted symmetry index is given as the arithmetic mean difference of fluorescence intensity (I) and occupancy (S) of Kar9 between the old (OP) and new pole (NP), normalized to total (T) occupancy and fluorescence:

$$WSI=mean\left(\frac{{S}_{OP}\cdot {I}_{OP}-{S}_{NP}\cdot {I}_{NP}}{{S}_{T}\cdot {I}_{T}}\right)$$

Given this expression, cells that are asymmetric old will have a WSI of 1, asymmetric new a WSI of − 1, and symmetric cells will have WSIs distributed between 1 and − 1 to reflect the occupancy and intensity of Kar9 signal at the biased pole.

### Spindle length

The spindle length is simply determined as the distance between the two spindle pole coordinates:

$${l}_{t}=\sqrt{{({x}_{O}-{x}_{N})}^{2}+{({y}_{O}-{y}_{N})}^{2}+{({z}_{O}-{z}_{N})}^{2}}$$

### Kar9 residence time in the bud

The mother-bud boundary is determined by the line defined by the two bud neck coordinates (x_1_, y_1_) and (x_2_, y_2_):

$$m=\frac{{y}_{2}-{y}_{1}}{{x}_{2}-{x}_{1}}$$

$$b= {y}_{1}-m{x}_{1}$$

$$y=mx+b$$

x,y coordinates of each Kar9 spot are then used to determine if the coordinates are within the bud region or not. Orientation of the bud is used to establish if the bud is above or below the line defined by the bud neck. For instance, if the polarity axis is between 0° and 180° (the bud is facing upward), the bud is above the line. The calculation is as follows:

Given the coordinates of Kar9 at a time t (x_k_, y_k_), the y-value corresponding to the x_k_ on the line of the bud neck is determined:

$$y=m{x}_{k}+b$$

The true Kar9 y coordinate y_k_ is compared to the calculated y to determine if it is above or below the line of the bud neck:

if y_k_ ≥ y, (x_k_, y_k_) is in the bud, otherwise y_k_ < y, (x_k_, y_k_) is in the mother.

The number of spots in the bud is recorded and normalized to the length of the acquisition. If the polarity axis is between 180° and 360° (the bud is facing downward), the inverse comparison relationship is used:

if y_k_ ≤ y, (x_k_, y_k_) is in the bud, otherwise y_k_ > y, ( x_k_, y_k_) is in the mother.

## Supplementary Information

Supplementary Information
