# Supplementary material for: Kar9 symmetry breaking alone is insufficient to ensure spindle alignment
Source: Sci Rep. 2021 Feb 19;11:4227. doi: 10.1038/s41598-021-83136-w (PMC7895971; doi:10.1038/s41598-021-83136-w)
Supplement: Supplementary file 1 — Supplementary Information [file 41598_2021_83136_MOESM1_ESM.pdf]

Supplementary Materials

# Kar9 symmetry breaking alone is insufficient to ensure spindle alignment

Miram Meziane, Rachel Genthial and Jackie Vogel

## Supplementary Materials

### Supplementary Table S1 – Strains used in this study

| Name | Genotype | Notes | Reference |
|---|---|---|---|
| YV2380 | *Spc42-mKate2-HIS; Kar9-mNeonGreen-NAT; Mat a; LYS(+); met15Δ0(-)* | WT; generated by previous lab member Yohann Faivre. | This study. |
| YV2679 | *Spc42-mKate2-HIS; kar9AA-mNeonGreen-NAT; Mat a; LYS(+); met15Δ0(-)* | *kar9AA* was tagged with mNeonGreen and mated to Spc42-mKate2-HIS by previous lab member Yohann Faivre. | Liakopoulos D, *et al.* (2003); This study |
| YV2412 | *swe1Δ-KAN; Spc42-mKate2-HIS; Kar9-mNeonGreen-NAT; Mat a; LYS(+); met15Δ0(-)* | Made by crossing YV2380 with *swe1Δ* from Invitrogen deletion collection; generated by previous lab member Yohann Faivre. | This study. |
| YV2771 | *swe1Δ-KAN; Spc42-mKate2-HIS; kar9AA-mNeonGreen-NAT; Mat a; LYS(+); met15Δ0(-)* | Made by crossing YV2679 with *swe1Δ* from Invitrogen deletion collection | This study. |

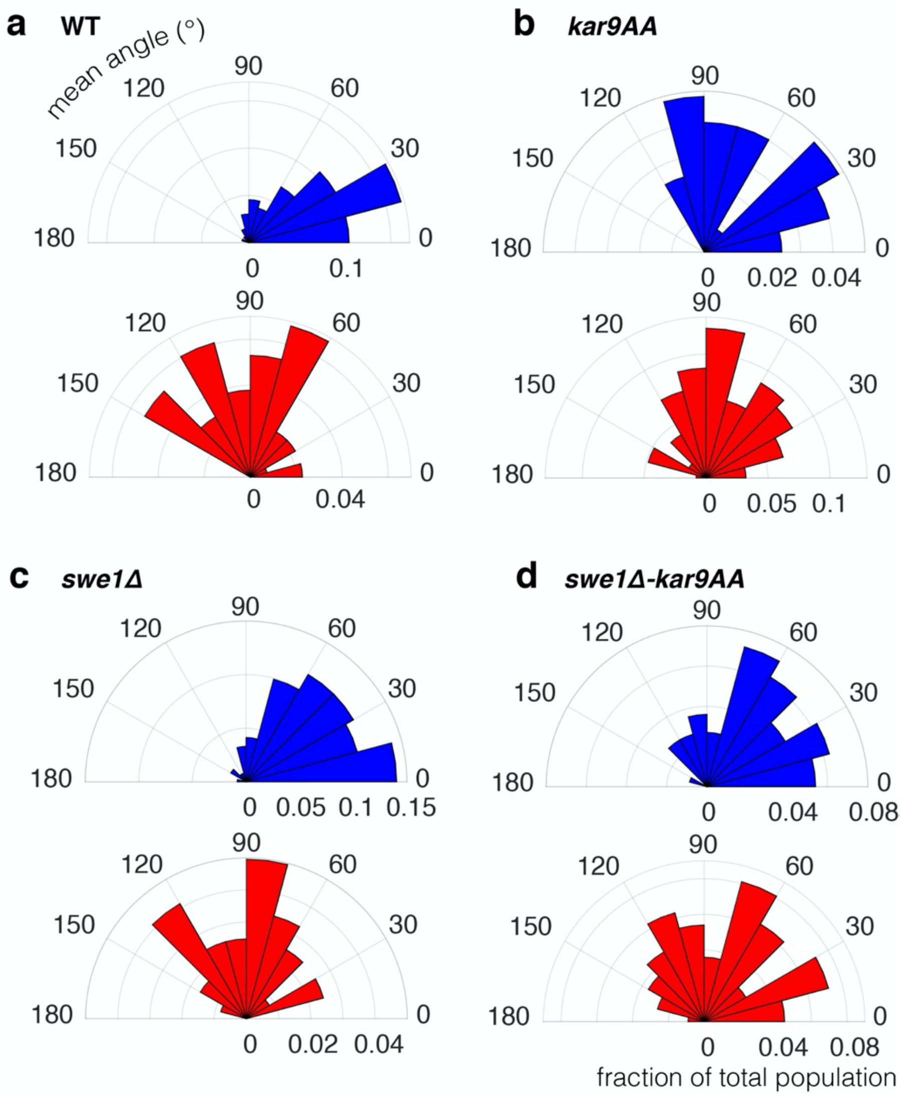


**Supplementary Figure 1. Mean 2D spindle alignment.** Symmetric spindles are unable to align themselves, regardless of genotype. (a) 2D alignment of WT spindles is biased toward small angles (proper alignment). (b) A subset of asymmetric kar9AA spindles are moderately biased toward small angles, while the others are clustered about misalignment (90$^o$). (c) Asymmetric *swe1Δ* spindles are biased toward short angles but less efficient than WT asymmetric spindles. (d) Similarly, asymmetric spindles of the double mutant are biased toward but less efficient at alignment. All are consistent with 3D measures described in the main text. Asymmetric spindles are in blue, symmetric ones in red.

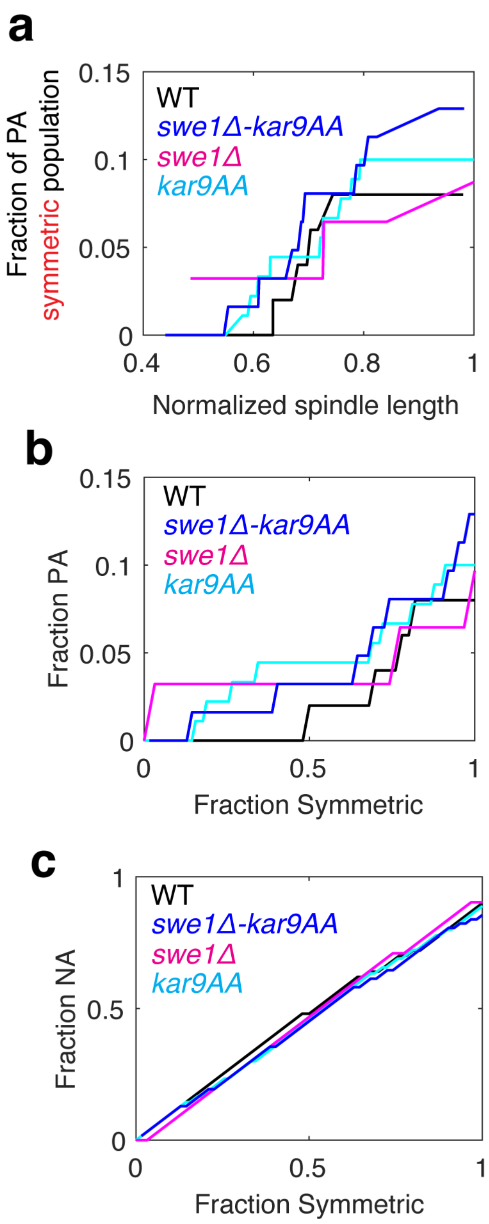


**Supplementary Figure S2. Changes in alignment as a function of spindle length and symmetry.** a) Fraction of perfectly aligned spindles that are symmetric as a function of increasing spindle length. Spindle length is normalized across different strains. b) Fraction of perfectly aligned spindles in the symmetric population across all conditions. Perfect alignment is similarly rare (0.08 – 0.15) across conditions. c) Spindle misalignment in the symmetric population. Misalignment is observed for the majority of symmetric spindles.

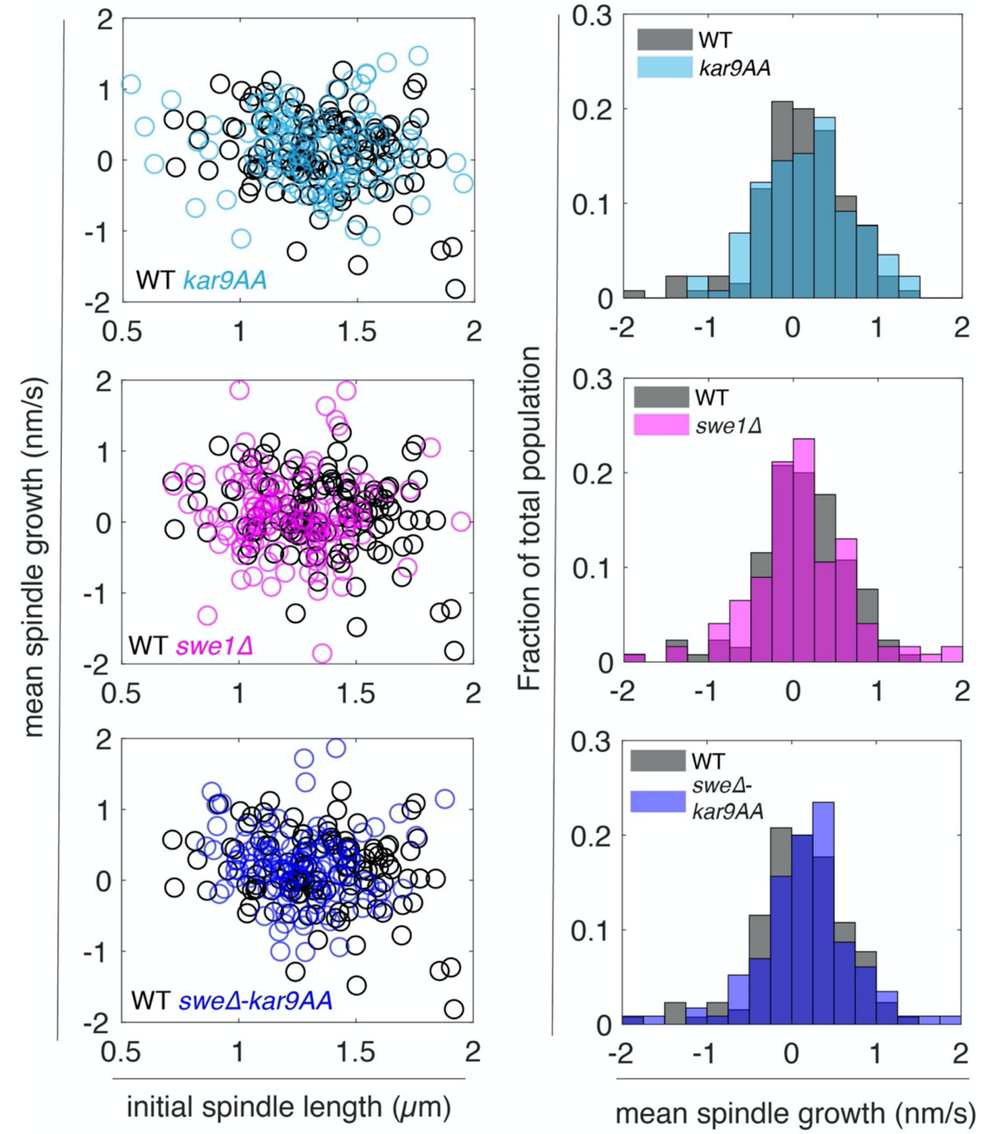


**Supplementary Figure S3. Spindle growth rate is unaltered by *kar9-AA*, *swe1Δ* and *swe1Δ; kar9-AA* mutations.** Mean spindle growth for WT (grey), *kar9-AA* (light blue), *swe1Δ* (pink) and *swe1Δ; kar9-AA* (blue). Distributions are comparable between strains. Spindle growth is independent of initial spindle length.
